# The Appeal of the Invalid Child

**Published:** 1921-04-30

**Authors:** 


### The Appeal of the Invalid Child.

The crowded annual meeting of the Invalid Children's Aid Association, held on April 26 in the large ballroom of Lady Violet Astor's house in Carlton House Terrace, testified to the interest taken in the work of this vigorous Association. Lord Queenborough, who presided, tersely put its needs as money, workers, and the continuance of the voluntary system. The first to pay off the present deficit of nearly £2,000, and to carry on and extend the work during the coming year; the second, to enable that work to be continued at its highest efficiency, and to secure the establishment of more branches; and the third, to maintain the bond between the classes of this country, which is the essence of the voluntary system. It is work such as that of the Invalid Children's Aid Association, said Lord Queenborough, which is the best bulwark against Bolshevism. Sir Napier Burnett, in a critical examination of the work and finances revealed by a study of the report, emphasised the value of the personal assistance rendered to each child by the Association; the seven residential homes had their beds occupied all the year round to the extent of 96 per cent.; whilst the exceeding economy of their management was achieved through the large volume of voluntary service each attracted. He referred with satisfaction to the co-ordination of this Association with the Central Committee for the Care of Cripples. The thirteen to fifteen bodies all concerned with the welfare of the child are to form part of the Central Institute of Infant and Child Welfare; the Carnegie Trustees have given £40,000 to buy central headquarters for the Institute, and No. 117 Piccadilly has been purchased, and will shortly be ready. In this co-ordination the work of the Invalid Children's Aid Association will remain the same—viz., personal service to the personal child. To put voluntary service generally to its best use its ranks should be closed by co-ordination. The Ministry of Health could do a great work in calling for the co-ordination of voluntary service with the view of conserving effort and preventing overlapping. He urged the importance of propaganda among children, and instanced its value by pointing to the number of personal gifts to the Association by children. He concluded by urging the workers and officials not to get disheartened by difficulties, for they are keeping open the gate to a fuller life for the little ones to pass. Dr. Sydney Fairbairn gave a brief but interesting account of the work, the progress of which could not, he said, be defined by statistics; it is beyond the power of figures to record the slow and steady improvement in the life and outlook of the sick and crippled child which the work of the Association effects. Father Bernard Vaughan and Miss Lena Ashwell appealed eloquently for funds, and dwelt upon the value, not only to the State but to the individual, of the voluntary system, and the personal service of the voluntary worker.

